# Circulating microRNA expression profiling and bioinformatics analysis of patients with coronary artery disease by RNA sequencing

**DOI:** 10.1002/jcla.23020

**Published:** 2019-09-05

**Authors:** Zhixiong Zhong, Wei Zhong, Qifeng Zhang, Qunji Zhang, Zhikang Yu, Heming Wu

**Affiliations:** ^1^ Center for Cardiovascular Diseases Meizhou People's Hospital (Huangtang Hospital), Meizhou Academy of Medical Sciences, Meizhou Hospital Affiliated to Sun Yat‐sen University Meizhou China; ^2^ Guangdong Provincial Key Laboratory of Precision Medicine, Clinical and Translational Research in Hakka Population Meizhou China; ^3^ Guangdong Provincial Engineering and Technology Research Center for Molecular Diagnostics of Cardiovascular Diseases Meizhou China; ^4^ Meizhou Municipal Engineering and Technology Research Center for Molecular Diagnostics of Cardiovascular Diseases Meizhou China; ^5^ Meizhou Municipal Engineering and Technology Research Center for Molecular Diagnostics of Major Genetic Disorders Meizhou China; ^6^ Center for Precision Medicine Meizhou People's Hospital (Huangtang Hospital), Meizhou Academy of Medical Sciences, Meizhou Hospital Affiliated to Sun Yat‐sen University Meizhou China

**Keywords:** Chinese, circulating microRNA, coronary artery disease, expression profiles, Hakka

## Abstract

**Background:**

MicroRNAs play a vital role in coronary artery disease. Abnormal expression of microRNAs has been found to be associated with the occurrence of CAD.

**Methods:**

We identified significantly differentially expressed microRNAs in plasma between 40 patients with CAD and 10 controls with NCA using RNA sequencing. The differentially expressed microRNAs were analyzed for Gene Ontology (GO) enrichment and Kyoto Encyclopedia of Genes and Genomes (KEGG) pathway enrichment.

**Results:**

Fifty cDNA libraries were constructed and sequenced, and a total of 1871.82 M raw reads were obtained, and 2135 microRNAs were found. Compared to the expressed microRNAs of NCA controls, 159 microRNAs were differentially expressed in CAD patients, including 119 upregulated microRNAs and 40 downregulated microRNAs. The top 10 upregulated miRNAs were miR‐144‐3p, miR‐34a‐5p, miR‐15b‐3p, miR‐22‐3p, miR‐29b‐3p, miR‐1270, miR‐6891‐5p, miR‐106a‐5p, miR‐15b‐5p, and hsa‐miR‐499b‐3p. The top ten downregulated miRNAs were miR‐4437, miR‐6842‐3p, miR‐4664‐3p, miR‐671‐3p, miR‐219a‐1‐3p, miR‐7848‐3p, miR‐664a‐3p, miR‐1284, miR‐361‐3p, and miR‐6780a‐5p. The target genes of differentially expressed microRNAs were related to many basic biological terms, such as biological process, cellular component, and molecular function. According to the KEGG pathway analysis, the most enriched pathways of the differentially expressed microRNAs were endocytosis, focal adhesion, axon guidance, and so on. Furthermore, six upregulated and two downregulated microRNAs were detected by qRT‐PCR (Quantitative Real‐time PCR) and ROC analysis for diagnosing CAD.

**Conclusion:**

The results suggest that the expression levels of some microRNAs may play a vital role in the physiological and pathological course of CAD. Our study may provide useful information for the diagnosis and treatment of CAD.

**Abbreviations**
ACS: and acute coronary syndrome
CAD: Coronary artery disease
CTnI: troponin I
ECG: electrocardiograph
GO: Gene ontology
HDL‐C: high‐density lipoprotein cholesterol
KEGG: Kyoto Encyclopedia of Genes and Genomes
LDL‐C: low‐density lipoprotein cholesterol
MiRNAs: microRNAs
NCA: normal coronary artery
NSTEMI: non‐ST‐segment elevation myocardial infarction
SA: stable angina
STEMI: ST‐segment elevation myocardial infarction
TG: triglycerides
UA: unstable angina

## 1 INTRODUCTION

Coronary artery disease (CAD) is a common heart disease. It is a myocardial malfunction and structural lesion caused by coronary artery stenosis and poor blood supply. The occurrence of CAD is closely related to the degree and number of coronary artery stenoses. Hypertension, diabetes, obesity, smoking, and drinking are the main factors that induce this disease.^1^, ^2^, ^3^ Clinically, it is often divided into stable angina (SA) and acute coronary syndrome (ACS). ACS can be divided into unstable angina (UA), acute non‐ST‐segment elevation myocardial infarction (NSTEMI) and acute ST‐segment elevation myocardial infarction (STEMI), according to severity. STEMI and NSTEMI are together known as acute myocardial infarction (AMI).^4^, ^5^

Coronary artery disease is one of the most fatal diseases in the world.^6^, ^7^ Atherosclerosis plays a pivotal role in the occurrence and progression of CAD. It is strongly associated with arterial stenosis because of the accumulation of atheromatous plaque, adipopexis, and extracellular matrix. Coronary thrombosis often leads to acute coronary events. Inflammation is involved in various stages of the formation and development of atherogenesis plaque, and inflammatory factors produced in this process are predicted to be potential markers of CAD. They play a key role in coordinating the interactions of the cells during the development of atherogenesis.^8^, ^9^, ^10^, ^11^ Inflammatory mediators involved in the function of some cells, including endothelial cells, macrophages, and T cells. They stimulate some signalling pathways in the onset and progression of atherosclerosis.^12^, ^13^ They can be regulated by microRNAs (miRNAs), which are small non‐coding RNAs that have emerged as important regulators in atherosclerosis.^14^, ^15^, ^16^, ^17^, ^18^

MicroRNAs are a class of single‐strand, non‐coding RNAs with a length of 18‐22 bp that play a vital regulatory role in different cell development processes by downregulating the expression of their target genes.^19^, ^20^ The analysis of the expression of microRNA in blood, tissue, or cell samples provides important information for the study of the biological functions of these molecules. In recent years, researchers have developed many methods to detect the differences in the expression of microRNAs in some physiological and pathological processes, and they have found that the abnormal expression of microRNAs is connected with the occurrence of cancer,^21^ nervous disorders,^22^, ^23^ diabetes,^24^ and heart diseases.^25^, ^26^, ^27^ Many studies have shown that microRNAs have some important regulatory effects in the cardiovascular system, including the heart, inflammatory response, angiogenesis, and metabolism.^20^, ^28^, ^29^, ^30^ Abnormal expression of microRNAs has been found to be related to the occurrence of CAD.

In this study, the differential expression of microRNAs in CAD patients and normal coronary artery (NCA) controls was detected by high‐throughput sequencing. We explored the role of microRNAs in CAD, and we further explored the relationship between microRNAs and the onset and progression of CAD to provide new ideas for the diagnosis and treatment of CAD.

## 2 MATERIALS AND METHODS

### 2.1 Study subjects

Between February 2016 and April 2017, angiographically confirmed CAD patients and NCA controls were prospectively enrolled in this study from the Department of Cardiovascular Diseases in Meizhou Peoples' Hospital. The diagnosis of CAD was based on the ACC/AHA classification. To be eligible, the patients had to have more than or equal to 50% diameter stenosis in the epicardial coronary arteries. Other inclusion criteria: chest pain, ischemic changes in electrocardiograph (ECG), and increased myocardial enzymes. All subjects underwent coronary angiography to confirm the diagnosis. Exclusion criteria: aortic dissection, ejection fraction less than 20%, pulmonary embolism, tumor, infectious or autoimmune diseases, vulnus, severe renal dysfunction (creatinine value >265 μmol/L), a recent surgical procedure, liver dysfunction, and blood‐borne infectious diseases, including HIV/AIDS, HBV, and HCV. Patients with myocarditis, pericarditis, and cardiomyopathy were also excluded.

Fifty subjects, 35 males and 15 females (2.3:1), were included in this study. They were classified into 5 groups: normal coronary artery (NCA), SA, UA, NSTEMI, and STEMI. The AMI patients presented ischemic chest pain, elevated levels of cardiac enzymes, and ST‐T changes. The UA patients had angina, with irregular angina at rest, and no rise in troponin. The SA patients had stable angina, lasting up to 10 minutes. In each group, two physicians independently confirmed the angiographic data. This study was approved by the Human Ethics Committees of Meizhou People's Hospital, Guangdong Province, China and followed the Helsinki Declaration. Informed consent was obtained from all subjects in this study.

### 2.2 Sample collection and processing

Two samples were taken before the coronary angiography surgery, one for the extraction of whole‐blood RNA and one for the detection of BNP and cTnI. Peripheral blood samples (6 mL) were taken from the anterior elbow vein using EDTA anticoagulant tubes, mixed gently up and down for 10 times, saved at 4°C, and subjected to plasma separation within 1 hour. The blood samples were centrifuged at 800 *g* for 10 minutes, and the plasma was transferred to a 1.5 mL centrifuge tube (RNase‐free). Total RNA was obtained with an RNeasy Kit (TianGene), and RNA integrity was assessed using an Agilent Bioanalyzer 2100 system (Agilent Technologies).

### 2.3 Library construction and high‐throughput sequencing

After screening test samples, a library of small RNAs was constructed using an Illumina TruSeq Small RNA Sample Prep Kit, which directly added joints to the small RNA using the 3' and 5' end special structures (5' end phosphate group, complete 3' end hydroxyl). The RNA was then reverse‐transcribed to cDNA. The target DNA fragments after PCR were separated by PAGE, and the cDNA libraries were recovered by recycling and purifying.

After the libraries were constructed, the libraries were preliminarily quantified by Qubit2.0 and diluted to 1 ng/µL. Then, the insert size of the libraries was detected by the Agilent 2100. If the insert size was in line with expectations, accurate quantification was carried out by qPCR to confirm the effective concentration of libraries (>2 nmol/L) to ensure the quality of the libraries. Sequencing libraries were constructed and confirmed, and the different libraries were sequenced according to the requirements, which was performed on an Illumina HiSeq 2500.

### 2.4 Identification of differentially expressed genes

The raw reads in FASTQ format were filtered to remove the adapter sequences, poly‐N sequences, and low‐quality sequences before data analysis. The remaining reads were also called “clean reads” and were acquired for transcriptome assembly and quantification. Next, the index of the reference genome was built using Bowtie v2.0.6, and paired‐end clean reads were mapped to the reference genome using TopHat v2.0.9. Transcriptome assemblies were generated using Cufflinks v2.1.1 with the default parameters. The microRNA sequence reads of each sample were normalized to fragments per kilobase of transcript per million mapped reads (FPKM) values using Cuffdiff v2.1.1. The *P* value and fold change were calculated for each gene. *P* < .05 and |log_2_(foldchange)|>1 were set as the combined threshold for significantly differential expression.

### 2.5 Gene ontology and KEGG enrichment analysis

Gene ontology analysis was carried out to determine the potential biological process terms of the differentially expressed genes in GO annotations.^31^ Pathway analysis was utilized to find the significant pathways of the differentially expressed genes according to the KEGG database.^32^ Fisher's exact test was applied to evaluate whether the GO terms or the KEGG pathways were enriched among the differentially expressed genes, and *P* < .05 was set as the threshold for.

### 2.6 Quantitative real‐time PCR

To verify the reliability of the RNA sequencing data, some microRNAs with differential expression were detected by qRT‐PCR. Total RNA in the plasma was extracted using an RNeasy Kit (TianGene) and was reverse‐transcribed to cDNA. qPCR was performed using the following conditions on the LightCycler 480 (Roche): 10 minutes at 95°C, then 40 cycles of amplification (2 seconds at 95°C for denaturation, 20 seconds at 60°C for annealing, and 10 seconds at 70°C for elongation). We verified the microRNA expression by qPCR using U6 snRNA as the internal control with the 2^‐ΔΔ^ *^C^* ^T^ method.

### 2.7 Statistical analysis

**Figure 1:**
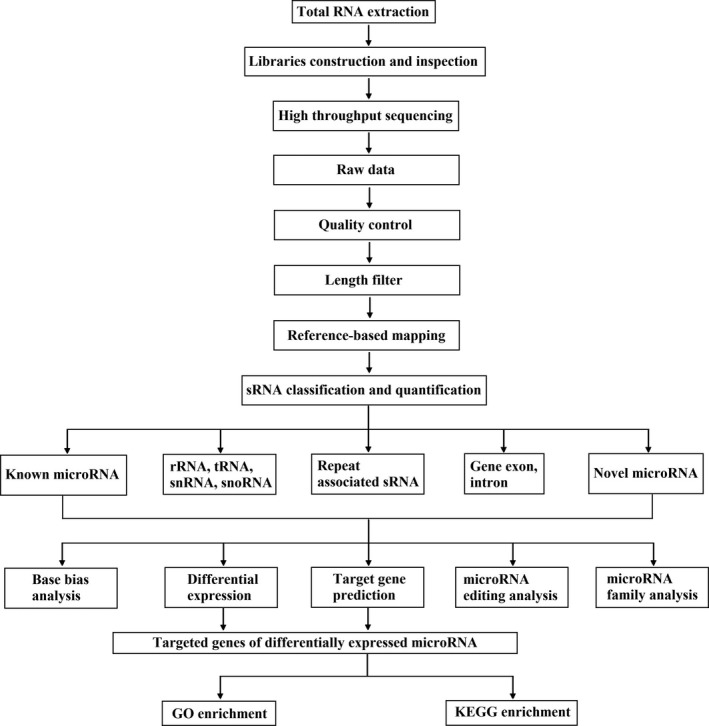
The technical route and methods of this study

Data analysis was performed in SPSS statistical software version 19.0 (International Business Machines Corporation). The mean ± SD was used to report the data. The chi‐square test and ANOVA were used for comparison between groups. *P* < .05 was considered statistically significant. The workflow of the experiment is shown in Figure 1.

## 3 RESULTS

### 3.1 Clinical characteristics of subjects

A total of 40 CAD patients and 10 NCA controls were recruited in the study. The clinical features of the 50 subjects in this study are shown in Table 1. There was a higher prevalence of diabetes and total cholesterol (TC) in the UA group than in the other groups (*P* = .0435 and *P* = .0045, respectively), whereas high‐density lipoprotein cholesterol (HDL‐C) level in the NSTEMI patients was significantly higher than in the other groups (*P* < .001). The cardiac troponin I (cTnI) levels in the UA, NSTEMI, and STEMI patients were significantly higher than in the NCA and SA groups (*P* < .001). There were no significant differences in age, sex, smoking, drinking, systolic BP, diastolic BP, hypertension, triglycerides (TG), or low‐density lipoprotein cholesterol (LDL‐C) between the CAD patients and NCA controls.

**Table 1:** The baseline clinical characteristics

Variable	NCA	SA	UA	NSTEMI	STEMI	*P* value
Age (y)	56.0 ± 8.5	62.7 ± 11.5	60.4 ± 7.2	54.3 ± 10.1	59.1 ± 9.2	.5989
Sex (male)	5 (50%)	6 (60%)	7 (70%)	6 (60%)	5 (50%)	.8864
Smoking	3 (30%)	1 (10%)	2 (20%)	2 (20%)	3 (30%)	.8031
Drinking	1 (10%)	0 (0%)	1 (10%)	0 (0%)	0 (0%)	.5371
Systolic BP (mm Hg)	132.4 ± 10.9	130.1 ± 14.2	129.4 ± 13.4	138.7 ± 16.8	124.2 ± 18.4	.9111
Diastolic BP (mm Hg)	85.1 ± 11.4	81.2 ± 12.5	77.3 ± 6.4	83.0 ± 10.9	79.3 ± 10.7	.3902
Hypertension	2 (20%)	3 (30%)	7 (70%)	4 (40%)	5 (50%)	.1936
Diabetes	1 (10%)	0 (0%)	4 (40%)	0 (0%)	3 (30%)	.0435
Hyperlipidemia	4 (40%)	1 (10%)	5 (50%)	5 (50%)	2 (20%)	.2081
TC, mmol/L	1.34 ± 0.57	1.17 ± 0.58	2.24 ± 0.87	1.94 ± 1.73	1.65 ± 1.13	.0045
TG, mmol/L	4.54 ± 0.86	4.46 ± 0.60	4.41 ± 0.61	5.57 ± 1.34	4.53 ± 0.97	.8640
HDL‐C, mmol/L	1.21 ± 0.41	1.24 ± 0.23	0.95 ± 0.21	1.30 ± 0.40	1.05 ± 0.07	.0083
LDL‐C, mmol/L	2.39 ± 0.53	2.53 ± 0.55	2.50 ± 0.52	3.32 ± 1.31	2.88 ± 1.02	.9018
cTnI, µg/L	0.013 ± 0.037	0.009 ± 0.025	2.268 ± 7.109	2.274 ± 4.428	5.945 ± 9.124	.0142
BNP, pg/L	392.56 ± 949.10	694.63 ± 1094.89	1590.44 ± 4760.54	1782.83 ± 3926.01	2579.71 ± 2950.41	.0874

Abbreviations: BNP, brain natriuretic peptide; cTnI, cardiac troponin I; HDL‐C, high‐density lipoprotein cholesterol; LDL‐C, low‐density lipoprotein cholesterol; TC, total cholesterol; TG, triglycerides.

### 3.2 Overview of the sequencing data

In this study, 50 cDNA libraries were constructed and sequenced. A total of 1871.82 M raw reads were obtained. We first removed low‐quality reads: the reads in which (a) more than 50% of bases had a Q value less than or equal to 5; (b) the unable‐to‐determine‐base‐information ratio was greater than 10%; (c) there was 5' joint contamination; (d) there was no 3' joint sequence or insert fragment; and (e) there was a polyA/T/G/C 3' flanking sequence. After their removal, the clean reads remained. The Q30 ranged between 90.77% and 98.93% for each sample, and more than 90.62% of the total clean reads were mapped. Generally, these results show that the quality of these libraries was good and suitable for analysis. Detailed sequencing data quality information is presented in Table S1 and Table S2.

### 3.3 Differentially expressed microRNAs in plasma

**Figure 2:**
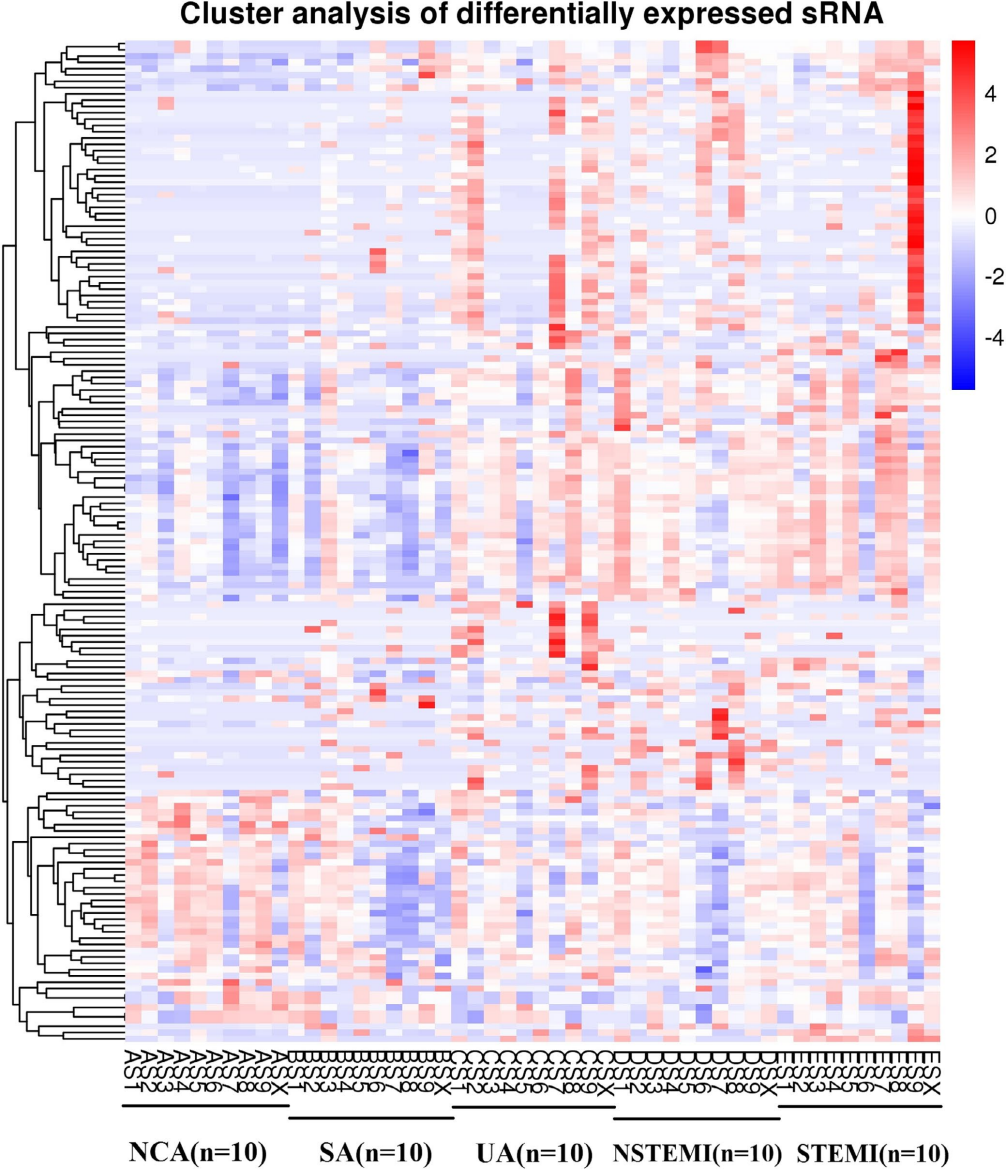
Hierarchical clustering of microRNAs in CAD patients and NCA controls. AS1‐ASX: NCA controls; BS1‐BSX: SA patients; CS1‐CSX: UA patients; DS1‐DSX: NSTEMI patients; ES1‐ESX: STEMI patients

MicroRNAs were analyzed with strict data quality control, and a total of 2135 microRNAs were found. To systematically study the level of microRNA expression associated with CAD, 40 patients with CAD and 10 NCA controls were analyzed in this study. The differences between the CAD patients and the NCA controls are shown in Figure 2. The blue and red in the picture indicate that the relative expression was reduced and raised, respectively.

**Figure 3:**
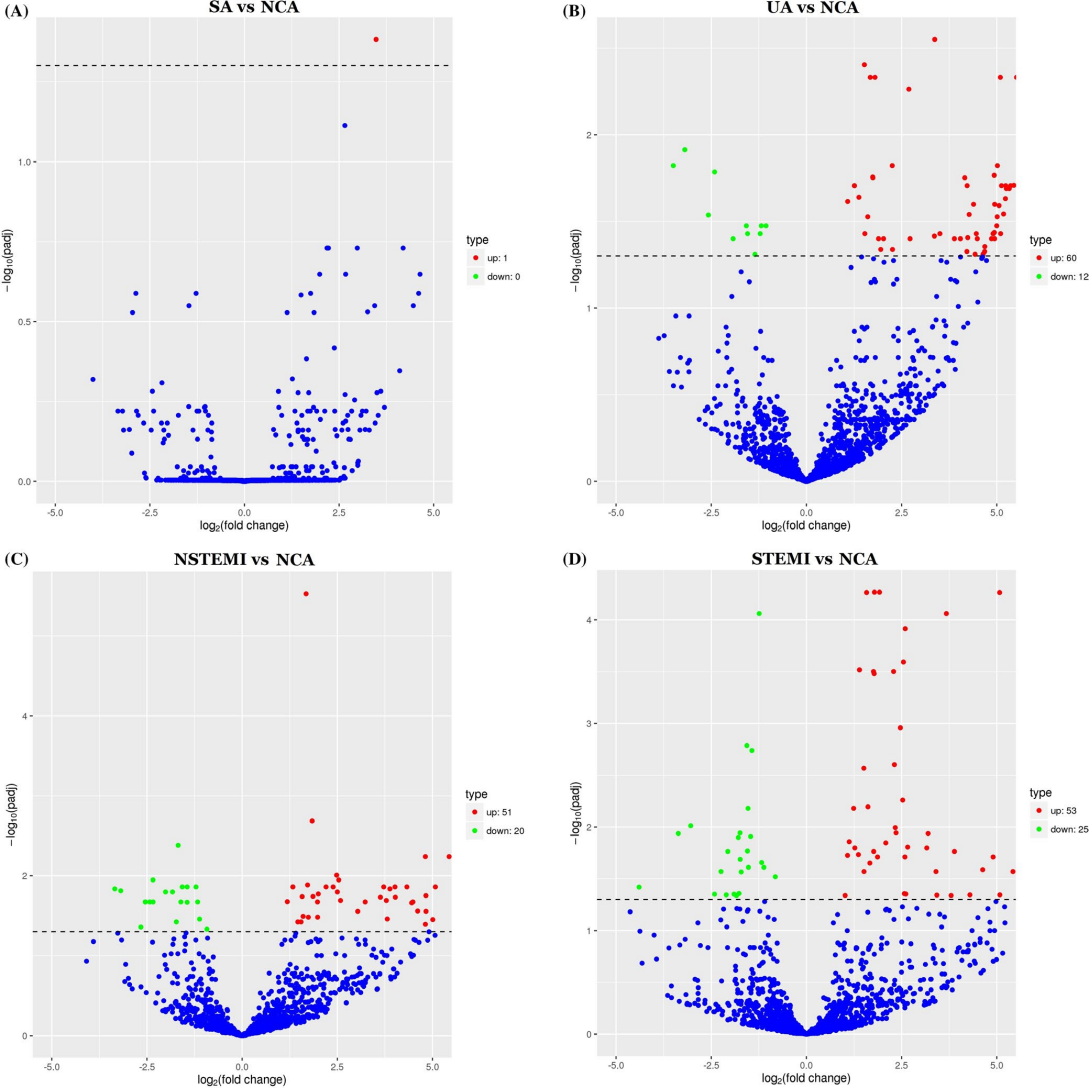
Volcano plot of differential microRNA expression. X‐axis: log_2_(fold change); Y‐axis: −1 × log_10_(corrected *q* value) for each probe. (Figure 3A: SA vs NCA; Figure 3B: UA vs NCA; Figure 3C: NSTEMI vs NCA; Figure 3D: STEMI vs NCA)

**Figure 4:**
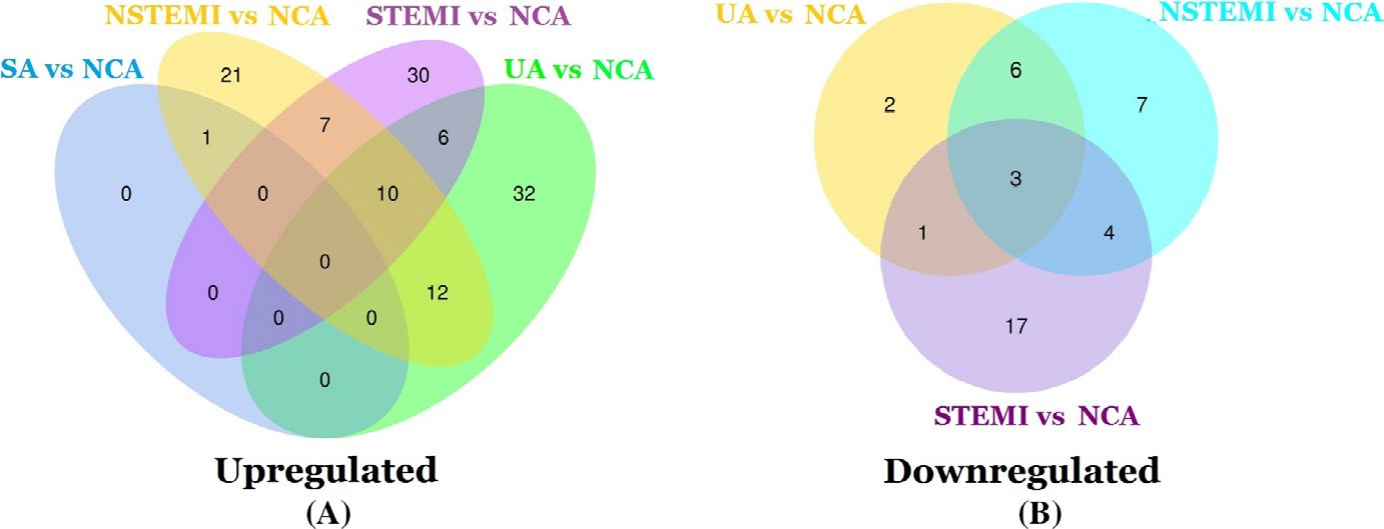
Venn diagrams of differentially upregulated microRNAs (A) and differentially downregulated microRNAs (B) in CAD patients

**Figure 5:**
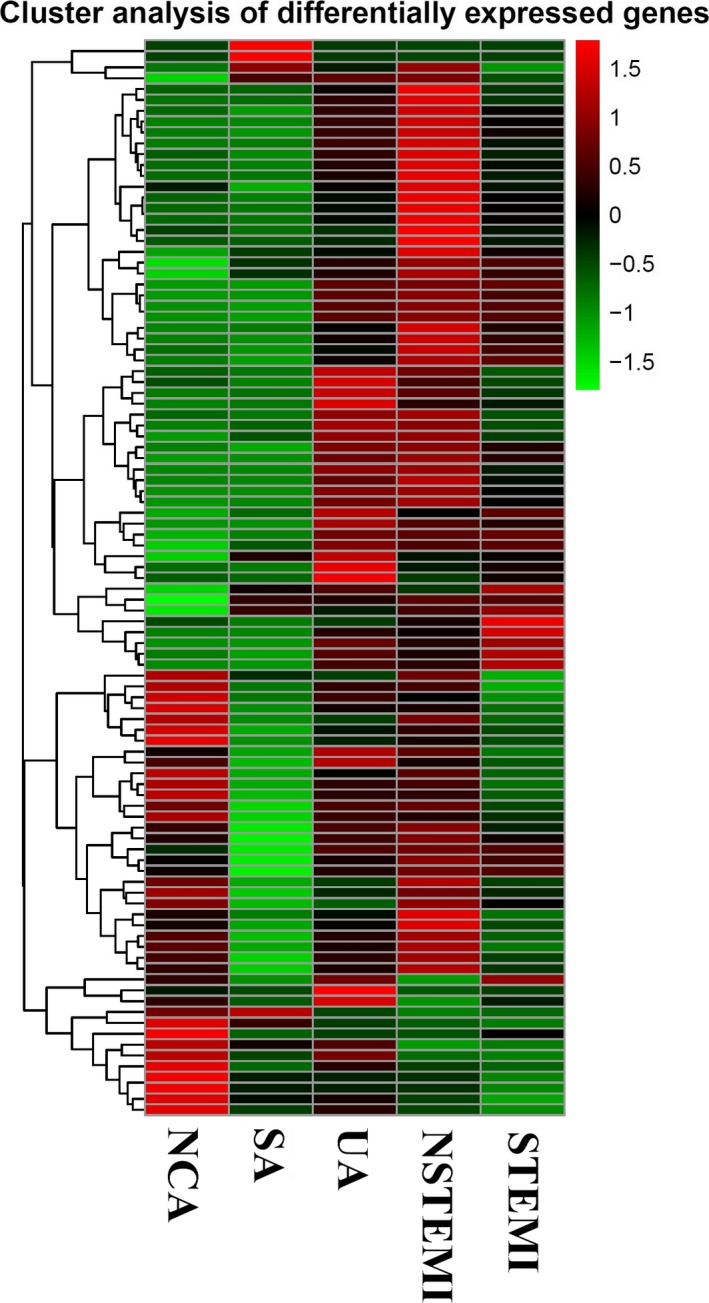
Hierarchical clustering of differentially expressed microRNAs in CAD patients and NCA controls. The red and green shades indicate upregulated and downregulated microRNAs, respectively, across all samples

Volcano plots were made to evaluate microRNAs between CAD patients and NCA controls (Figure 3). Compared to NCA, 159 differentially expressed microRNAs were discriminated in CAD patients, including 119 upregulated microRNAs and 40 downregulated microRNAs (*P* < .05). The top 10 upregulated miRNAs were miR‐144‐3p, miR‐34a‐5p, miR‐15b‐3p, miR‐22‐3p, miR‐29b‐3p, miR‐1270, miR‐6891‐5p, miR‐106a‐5p, miR‐15b‐5p, and hsa‐miR‐499b‐3p. The top ten downregulated miRNAs were miR‐4437, miR‐6842‐3p, miR‐4664‐3p, miR‐671‐3p, miR‐219a‐1‐3p, miR‐7848‐3p, miR‐664a‐3p, miR‐1284, miR‐361‐3p, and miR‐6780a‐5p. In the SA group, UA group, NSTEMI group, and STEMI group, there were 1, 60, 51, and 53 upregulated microRNA/microRNAs compared with NCA controls, respectively. There were 0, 12, 20, and 25 downregulated microRNAs compared with NCA controls, respectively. The details are shown in Venn diagrams of upregulated and downregulated differentially expressed microRNAs in CAD patients (Figure 4). Moreover, a horizontal comparison of microRNA expression was also performed at the overall level, and the trend changes of the altered expression profiles of 99 microRNAs were in accordance with the extent of CAD, as shown in Figure 5.

### 3.4 Gene ontology and KEGG pathway analyses

Gene ontology and KEGG pathway analysis showed that genes were sorted by hierarchical categories according to biological process, cellular component, and molecular function.

**Figure 6:**
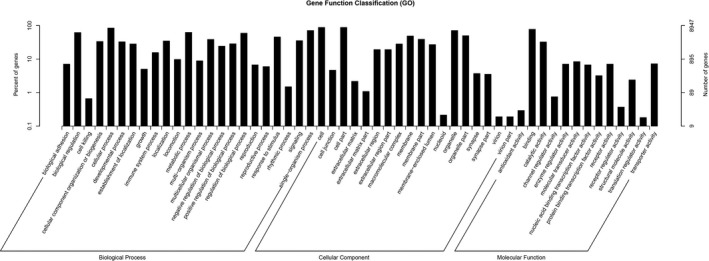
GO analysis of differentially expressed microRNAs covering three domains: biological process, cellular component, and molecular function. X‐axis: GO terms of biological process, cellular component, and molecular function. The green column indicates biological process, the red column indicates cellular component, and the blue column indicates molecular function. Y‐axis on the left: number of genes (microRNAs)

**Figure 7:**
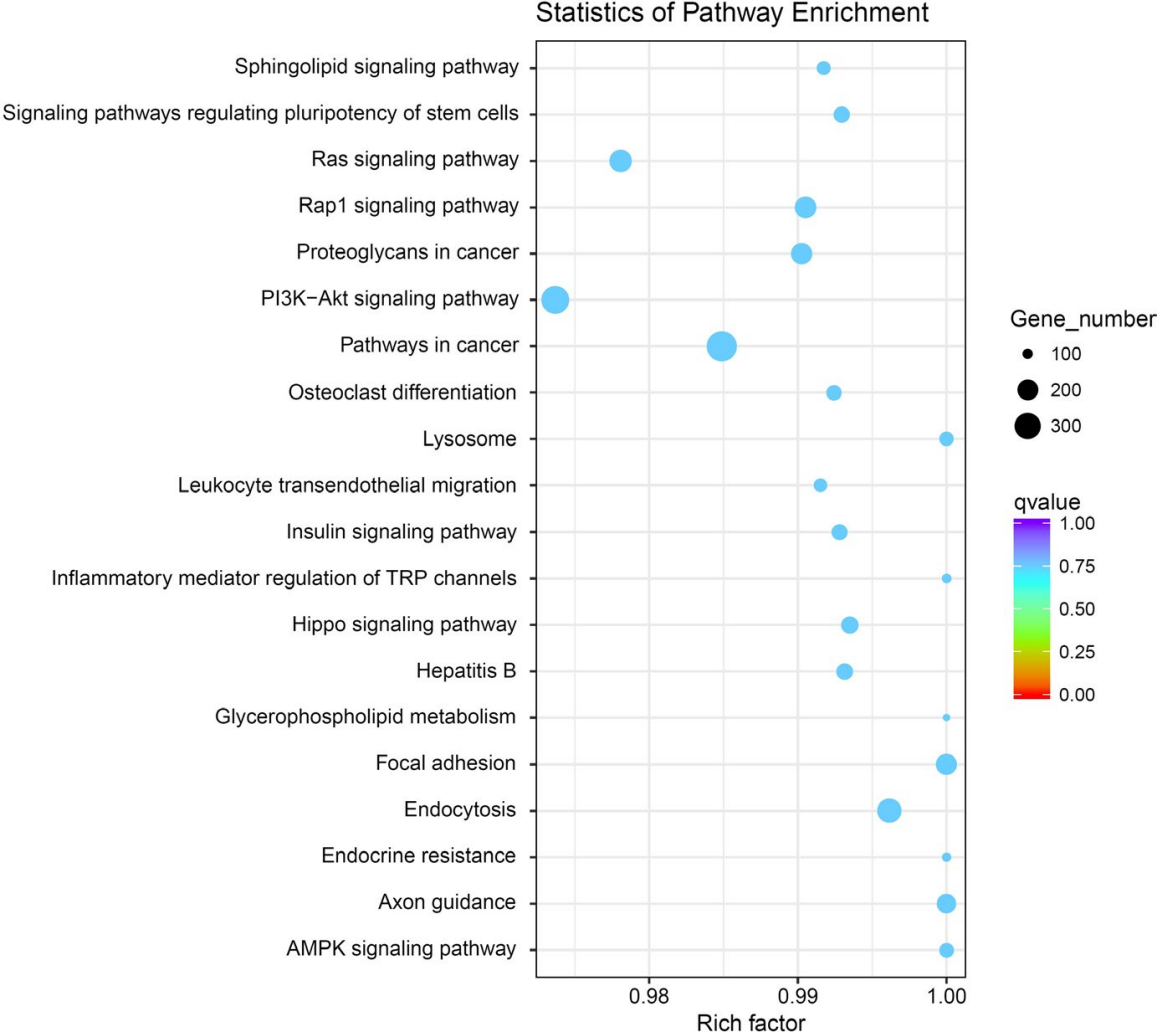
Pathway analysis of differentially expressed microRNAs. Pathway analysis is a functional analysis mapping genes to KEGG pathways and other pathway databases. The lower the *P* value, the more significant the pathway association

Among the identified mRNAs, 19 243 genes were related to biological function (Figure 6). The enriched GO terms of these potential targets included many biological events. The most enriched pathways related to the microRNAs with differential expression were endocytosis, pathways in cancer, focal adhesion, axon guidance, and so on, according to the KEGG pathway analysis (Figure 7). The detailed information on the GO analysis and the top 20 pathways according *P* value in the KEGG enrichment analysis are shown in Table S3 and Table S4, respectively.

### 3.5 qRT‐PCR validation of microRNA expression

**Figure 8:**
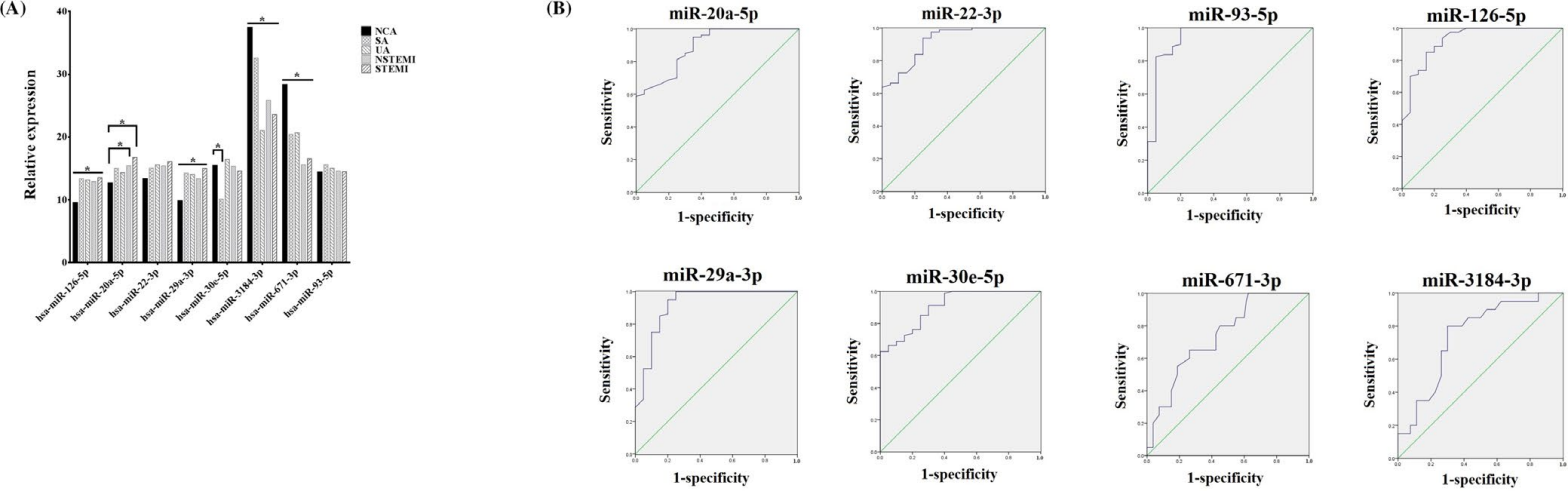
Validation of RNA‐seq results by using quantitative qRT‐PCR and ROC analysis

To confirm the sequencing data of microRNA expression levels, six upregulated microRNAs (has‐miR‐126‐5p, has‐miR‐20a‐5p, has‐miR‐22‐3p, has‐miR‐29a‐3p, has‐miR‐30e‐5p, and has‐miR‐93‐5p) and two downregulated microRNAs (has‐miR‐3184‐3p and has‐miR‐671‐3p) were randomly selected. We confirmed the microRNA expression in the plasma of CAD patients (n = 80) and NCA controls (n = 20) by qRT‐PCR using U6 snRNA as the internal control with the 2^‐ΔΔCT^ method. Differences in the expression of all eight microRNAs were detected in CAD patients compared with NCA controls, and the results of qRT‐PCR were consistent with those of RNA sequencing analysis (Figure 8A).

### 3.6 ROC analysis

ROC analysis was done to evaluate the diagnostic value of some differentially expressed microRNAs that were randomly selected for differentiating between CAD patients and NCA controls. MicroRNAs with *P* < .05 and AUC > 0.6 were selected as potential markers to distinguish CAD patients and NCA controls (Figure 8B). The ROC curves yielded the following AUCs: miR‐20a‐5p (AUC = 0.892, upregulated), miR‐22‐3p (AUC = 0.924, upregulated), miR‐29a‐3p (AUC = 0.920, upregulated), miR‐30e‐5p (AUC = 0.907, upregulated), miR‐93‐5p (AUC = 0.944, upregulated), miR‐126‐5p (AUC = 0.928, upregulated), miR‐671‐3p (AUC = 0.739, downregulated), and miR‐3184‐3p (AUC = 0.741, downregulated), which were found to distinguish CAD patients from NCA subjects.

## 4 DISCUSSION

Heart disease is the main cause of death for both males and females and is a great burden to some countries, which includes the cost of health care services, medications, and lost productivity.^33^, ^34^, ^35^, ^36^ Before vessel obstruction, the vascular wall has a plaque for a long time, and several cytokines are involved in all stages of the formation and progression of atherogenic plaque, which provides a possibility for the prediction and early diagnosis of cardiovascular adverse events. There is a lack of good biomarkers that could be used in early clinical diagnosis, clinical risk stratification, and evaluation of the prognosis of CAD, and there is a great clinical demand for reliable non‐invasive biomarkers for CAD.^37^, ^38^

Many studies have found that microRNAs are important regulators of the onset and progression of coronary artery disease. In addition, some reports have shown that some microRNAs can be used as diagnostic or prognostic markers for CAD. MicroRNAs are a novel group of conserved non‐coding, single‐strand small RNAs that regulate gene expression after transcription, leading to translation inhibition or mRNA degradation.^39^ Many studies have shown that microRNAs participate in many biological processes and speculate that they regulate the expression of 1/3 or more of the genes in animals.^40^, ^41^ MicroRNAs are not only widely involved in important life processes, such as the normal growth, development, and metabolism of the body, but they also play an important regulatory role in the onset and progression of many diseases. Plasma or serum microRNAs can be biological indicators for early clinical diagnosis, and further exploration of microRNAs in the pathogenesis of some diseases has gotten increasing attention globally. These molecules provide a new research field to study early clinical diagnosis, clinical risk‐stratified diagnosis, and evaluation of the prognosis of CAD.

We reviewed the relevant literature on microRNAs related to cardiovascular disease and compared the previous results with ours. One study showed that miR‐126‐5p was not significantly upregulated or downregulated in CAD patients, but the level of miR‐126‐5p was significantly increased in patients whose LDL cholesterol was high.^42^ Another study indicated that circulating miR‐126‐5p is a potential biomarker for predicting type 2 diabetes and diabetic CAD.^43^ One study showed that the level of miR‐146a/b was observably increased in the CAD group compared with the non‐CAD group.^44^ The SNP in miR‐146a and rs2910164 G>C was significantly associated with an increased risk for CAD.^45^ Another report has shown that early growth response 2 (EGR2) is regulated by 8 microRNAs, including miR‐150. It might play important roles in patients before and after off‐pump coronary artery bypass (OPCAB) surgery via the regulation of associated genes.^46^ Many studies have shown that miR‐17‐5p is an important regulator in the G1/S phase cell cycle transition.^47^ miR‐17‐5p is associated with breast cancer,^48^, ^49^ lung cancer,^50^ liver cancer,^51^ gastric cancer,^52^ pancreatic cancer,^53^ and multiple sclerosis^54^ and may have a role in the onset and progression of heart failure.^55^ In the present study, we found that miR‐17‐5p was significantly upregulated in CAD patients. MiR‐181a‐2‐3p has been associated with cerebral cavernous malformations^56^ and follicular variant of papillary thyroid carcinoma (FVPTC).^57^ Another study showed that rs174545 (FADS1: miR‐181a‐2), affecting a microRNA‐binding site, can affect microRNA‐mediated regulation of cardiometabolic genes.^58^ Our results showed that miR‐181a‐2‐3p was significantly downregulated in CAD patients. One study showed that miR‐181a‐5p and miR‐181b‐5p were upregulated in congestive heart failure, and these microRNAs may play a role in the onset and progression of this disease, as they might be related to pathways associated with disease progression.^55^ A previous study showed that acute heart failure and renal disease were correlated with obviously decreased levels of miR‐199a‐3p and miR‐423‐5p compared with healthy controls.^59^ In this study, we found that miR‐199a‐3p was significantly upregulated in CAD patients and that miR‐423‐5p was downregulated. A previous study showed that reduced miR‐214‐3p expression may contribute to MEF2C expression in myocardial hypertrophy.^60^ Some studies have shown that circulating miR‐22‐3p contains important information about chronic heart failure patients^61^ and participates in regulating the expression of hypertension‐related genes and in the development of hypertension.^62^ In our research, we excluded patients with heart failure, and circulating miR‐22‐3p was significantly upregulated in CAD patients compared with NCA controls. These results show that miR‐22‐3p may contain important information on the onset, progression, prognosis, and other aspects in patients with cardiovascular disease. A previous study indicated that miR‐26b‐5p was upregulated in the serum of left ventricular hypertrophy (LVH) hypertensive patients compared with healthy controls.^63^ In our research, circulating miR‐26b‐5p was significantly upregulated in CAD patients. A previous study indicated that miR‐30a‐5p and miR‐30e‐5p may have a role in the onset and progression of heart failure.^55^ In our research, circulating miR‐30a‐5p and miR‐30e‐5p were significantly upregulated in CAD patients.

Our results suggested the importance of some microRNAs that have not been reported in the study of cardiovascular disease, including the upregulated miR‐100‐5p, miR‐107, miR‐1‐3p, miR‐152‐3p, miR‐16‐5p, miR‐185‐5p, miR‐186‐5p, miR‐20a‐5p, miR‐24‐3p, miR‐27a‐3p, miR‐29a‐3p, miR‐3074‐5p, miR‐340‐5p, miR‐363‐3p, miR‐425‐5p, miR‐451a, miR‐485‐3p, and miR‐93‐5p and the downregulated miR‐1228‐5p, miR‐1246, miR‐1273h‐3p, miR‐3184‐3p, miR‐589‐5p, miR‐671‐3p, miR‐6772‐3p, miR‐6842‐3p, and miR‐99b‐5p. They may also be used as potential biological markers for CAD. Interestingly, we found three novel microRNAs, miR‐3184‐3p, miR‐451a, and miR‐6772‐3p. The research on the functions of these microRNAs will be the focus of our next work.

The enriched GO terms of these potential target genes related to the differentially expressed microRNAs involved some basic biological processes, such as intracellular, extracellular vesicle, and protein‐DNA complex assembly. In KEGG pathway analysis, the most enriched pathways among the differentially expressed microRNAs were endocytosis, pathways in cancer, focal adhesion, axon guidance, and so on. These microRNAs may have a role in the pathways related to disease progression.

Our study was on the microRNA expression profiles in the Chinese Hakka population, but there are still some shortcomings to our research. The number of subjects in this study was low. In addition, there may be differences in the results of patients from different regions and races. Therefore, these results need to be verified in a larger number of people. Again, the functions of these microRNAs have not yet been determined. More detailed studies are needed to determine the biological effects of the microRNAs.

## 5 CONCLUSIONS

This study comprehensively identified and analyzed microRNA expression in CAD patients using RNA sequencing. The results suggested that the expression levels of some microRNAs may play a vital role in the onset and course of progression of CAD. This discovery might provide useful information for the diagnosis and treatment of CAD. The function of the corresponding microRNAs will be the focus of our next work.

## AUTHOR CONTRIBUTIONS

Zhixiong Zhong, Heming Wu, and Wei Zhong designed the study. Zhixiong Zhong and Heming Wu performed the experiments. Wei Zhong and Qifeng Zhang recruited subjects and collected clinical data. Qunji Zhang and Zhikang Yu helped to analyze the data. Heming Wu prepared the manuscript. All authors were responsible for critical revisions, and all authors read and approved the final version of this work.

## Data Availability

The data used to support the findings of this study are available from the corresponding author upon request.
