# Supplementary material for: Circulating microRNA expression profiling and bioinformatics analysis of patients with coronary artery disease by RNA sequencing
Source: J Clin Lab Anal. 2019 Sep 5;34(1):e23020. doi: 10.1002/jcla.23020 (PMC6977390; doi:10.1002/jcla.23020)
Supplement: Supplementary file 1 [file JCLA-34-e23020-s001.docx]

**TableS1 Data output quality of samples**

| Sample | Reads | Bases | Error rate | Q20 | Q30 | GC content |
| --- | --- | --- | --- | --- | --- | --- |
| NCA-1 | 47837000 | 2.392G | 0.01% | 99.24% | 98.17% | 53.96% |
| NCA-2 | 46517000 | 2.326G | 0.01% | 99.33% | 98.33% | 54.01% |
| NCA-3 | 42768000 | 2.138G | 0.01% | 99.03% | 97.59% | 53.14% |
| NCA-4 | 30975000 | 1.549G | 0.01% | 98.98% | 97.55% | 53.05% |
| NCA-5 | 28213000 | 1.411G | 0.01% | 99.28% | 98.19% | 53.21% |
| NCA-6 | 29336000 | 1.467G | 0.01% | 99.40% | 98.43% | 53.53% |
| NCA-7 | 34671000 | 1.734G | 0.01% | 99.08% | 97.85% | 53.47% |
| NCA-8 | 37756108 | 1.888G | 0.01% | 99.02% | 97.77% | 54.00% |
| NCA-9 | 28790554 | 1.440G | 0.01% | 99.22% | 98.18% | 53.66% |
| NCA-10 | 43153000 | 2.158G | 0.01% | 98.87% | 97.35% | 53.58% |
| SA-1 | 35515000 | 1.776G | 0.01% | 99.35% | 98.42% | 53.94% |
| SA-2 | 28115000 | 1.406G | 0.01% | 99.15% | 97.95% | 53.88% |
| SA-3 | 29348000 | 1.467G | 0.01% | 99.21% | 98.08% | 53.25% |
| SA-4 | 44099317 | 2.205G | 0.01% | 99.16% | 97.95% | 53.75% |
| SA-5 | 33895000 | 1.695G | 0.01% | 98.78% | 97.14% | 53.40% |
| SA-6 | 31840000 | 1.592G | 0.01% | 99.10% | 97.91% | 53.41% |
| SA-7 | 49882000 | 2.494G | 0.01% | 98.79% | 96.93% | 51.43% |
| SA-8 | 32456000 | 1.623G | 0.01% | 97.82% | 94.96% | 53.61% |
| SA-9 | 29552000 | 1.478G | 0.01% | 98.78% | 97.25% | 53.46% |
| SA-10 | 30337124 | 1.517G | 0.01% | 95.97% | 90.77% | 54.00% |
| UA-1 | 33231000 | 1.662G | 0.01% | 99.44% | 98.68% | 52.88% |
| UA-2 | 41508000 | 2.075G | 0.01% | 98.97% | 97.64% | 52.36% |
| UA-3 | 34452000 | 1.723G | 0.01% | 99.42% | 98.71% | 54.07% |
| UA-4 | 30737000 | 1.537G | 0.01% | 99.40% | 98.66% | 53.80% |
| UA-5 | 28222000 | 1.411G | 0.01% | 99.19% | 98.16% | 53.94% |
| UA-6 | 37082000 | 1.854G | 0.01% | 99.37% | 98.55% | 53.49% |
| UA-7 | 28029000 | 1.401G | 0.01% | 99.13% | 97.99% | 53.27% |
| UA-8 | 47179000 | 2.359G | 0.01% | 99.33% | 98.45% | 52.73% |
| UA-9 | 29171000 | 1.459G | 0.01% | 99.55% | 98.93% | 53.48% |
| UA-10 | 28919000 | 1.446G | 0.01% | 99.43% | 98.57% | 53.22% |
| NSTEMI-1 | 49843000 | 2.492G | 0.01% | 99.34% | 98.37% | 53.14% |
| NSTEMI-2 | 41927000 | 2.096G | 0.01% | 99.28% | 98.23% | 53.15% |
| NSTEMI-3 | 52786000 | 2.639G | 0.01% | 99.45% | 98.74% | 53.35% |
| NSTEMI-4 | 48452000 | 2.423G | 0.01% | 99.37% | 98.46% | 53.53% |
| NSTEMI-5 | 41817000 | 2.091G | 0.01% | 99.44% | 98.69% | 53.82% |
| NSTEMI-6 | 43168000 | 2.158G | 0.01% | 98.88% | 97.41% | 51.65% |
| NSTEMI-7 | 49243000 | 2.462G | 0.01% | 98.25% | 95.91% | 52.42% |
| NSTEMI-8 | 43171723 | 2.159G | 0.01% | 98.82% | 97.38% | 52.89% |
| NSTEMI-9 | 51332000 | 2.567G | 0.01% | 99.28% | 98.32% | 53.85% |
| NSTEMI-10 | 40134973 | 2.007G | 0.01% | 99.32% | 98.48% | 54.02% |
| STEMI-1 | 37884000 | 1.894G | 0.01% | 99.27% | 98.33% | 53.36% |
| STEMI-2 | 32041078 | 1.602G | 0.01% | 99.46% | 98.79% | 53.01% |
| STEMI-3 | 45684819 | 2.284G | 0.01% | 99.21% | 98.28% | 53.77% |
| STEMI-4 | 32825000 | 1.641G | 0.01% | 98.80% | 97.27% | 53.63% |
| STEMI-5 | 42398842 | 2.120G | 0.01% | 99.30% | 98.47% | 53.70% |
| STEMI-6 | 44943000 | 2.247G | 0.01% | 99.35% | 98.47% | 53.39% |
| STEMI-7 | 29796000 | 1.490G | 0.01% | 99.01% | 97.67% | 53.64% |
| STEMI-8 | 28193000 | 1.410G | 0.01% | 99.42% | 98.66% | 53.42% |
| STEMI-9 | 28395000 | 1.420G | 0.01% | 97.68% | 94.65% | 51.19% |
| STEMI-10 | 34198000 | 1.710G | 0.01% | 99.41% | 98.62% | 53.51% |
