# Supplementary material for: Circulating microRNA expression profiling and bioinformatics analysis of patients with coronary artery disease by RNA sequencing
Source: J Clin Lab Anal. 2019 Sep 5;34(1):e23020. doi: 10.1002/jcla.23020 (PMC6977390; doi:10.1002/jcla.23020)
Supplement: Supplementary file 2 [file JCLA-34-e23020-s002.docx]

**TableS2 Categorization of reads and basic characteristics of samples**

| Sample | total reads | N% > 10% | low quality | 5 adapter contamine | 3 adapter null or insert null | with ployA/T/G/C | clean reads |
| --- | --- | --- | --- | --- | --- | --- | --- |
| NCA-1 | 47837000 (100.00%) | 292 (0.00%) | 0 (0.00%) | 20240 (0.04%) | 2259369 (4.72%) | 12571 (0.03%) | 45544528 (95.21%) |
| NCA-2 | 46517000 (100.00%) | 345 (0.00%) | 0 (0.00%) | 5160 (0.01%) | 1666430 (3.58%) | 16344 (0.04%) | 44828721 (96.37%) |
| NCA-3 | 42768000 (100.00%) | 192 (0.00%) | 0 (0.00%) | 21511 (0.05%) | 6582919 (15.39%) | 20137 (0.05%) | 36143241 (84.51%) |
| NCA-4 | 30975000 (100.00%) | 370 (0.00%) | 0 (0.00%) | 10656 (0.03%) | 2832753 (9.15%) | 19195 (0.06%) | 28112026 (90.76%) |
| NCA-5 | 28213000 (100.00%) | 222 (0.00%) | 0 (0.00%) | 11539 (0.04%) | 1225849 (4.34%) | 5280 (0.02%) | 26970110 (95.59%) |
| NCA-6 | 29336000 (100.00%) | 179 (0.00%) | 0 (0.00%) | 17894 (0.06%) | 1947142 (6.64%) | 9098 (0.03%) | 27361687 (93.27%) |
| NCA-7 | 34671000 (100.00%) | 291 (0.00%) | 0 (0.00%) | 106022 (0.31%) | 3626846 (10.46%) | 14535 (0.04%) | 30923306 (89.19%) |
| NCA-8 | 37756108 (100.00%) | 605 (0.00%) | 0 (0.00%) | 44343 (0.12%) | 1385809 (3.67%) | 5580 (0.01%) | 36319771 (96.20%) |
| NCA-9 | 28790554 (100.00%) | 486 (0.00%) | 0 (0.00%) | 28370 (0.10%) | 684850 (2.38%) | 4079 (0.01%) | 28072769 (97.51%) |
| NCA-10 | 43153000 (100.00%) | 944 (0.00%) | 0 (0.00%) | 81186 (0.19%) | 1654527 (3.83%) | 14272 (0.03%) | 41402071 (95.94%) |
| SA-1 | 35515000 (100.00%) | 477 (0.00%) | 0 (0.00%) | 18352 (0.05%) | 601112 (1.69%) | 6035 (0.02%) | 34889024 (98.24%) |
| SA-2 | 28115000 (100.00%) | 1126 (0.00%) | 0 (0.00%) | 35770 (0.13%) | 1440440 (5.12%) | 19458 (0.07%) | 26618206 (94.68%) |
| SA-3 | 29348000 (100.00%) | 181 (0.00%) | 0 (0.00%) | 2791 (0.01%) | 1498541 (5.11%) | 13615 (0.05%) | 27832872 (94.84%) |
| SA-4 | 44099317 (100.00%) | 377 (0.00%) | 0 (0.00%) | 27278 (0.06%) | 2666303 (6.05%) | 14593 (0.03%) | 41390766 (93.86%) |
| SA-5 | 33895000 (100.00%) | 755 (0.00%) | 0 (0.00%) | 26179 (0.08%) | 2037536 (6.01%) | 11589 (0.03%) | 31818941 (93.88%) |
| SA-6 | 31840000 (100.00%) | 1823 (0.01%) | 0 (0.00%) | 611950 (1.92%) | 2013909 (6.33%) | 15851 (0.05%) | 29196467 (91.70%) |
| SA-7 | 49882000 (100.00%) | 120 (0.00%) | 0 (0.00%) | 150475 (0.30%) | 23222490 (46.55%) | 17882 (0.04%) | 26491033 (53.11%) |
| SA-8 | 32456000 (100.00%) | 1709 (0.01%) | 0 (0.00%) | 767054 (2.36%) | 5108471 (15.74%) | 18293 (0.06%) | 26560473 (81.84%) |
| SA-9 | 29552000 (100.00%) | 1559 (0.01%) | 0 (0.00%) | 360140 (1.22%) | 4198519 (14.21%) | 14266 (0.05%) | 24977516 (84.52%) |
| SA-10 | 30337124 (100.00%) | 1210 (0.00%) | 0 (0.00%) | 24408 (0.08%) | 16339303 (53.86%) | 4352 (0.01%) | 13967851 (46.04%) |
| UA-1 | 33231000 (100.00%) | 802 (0.00%) | 0 (0.00%) | 7938 (0.02%) | 5001248 (15.05%) | 13773 (0.04%) | 28207239 (84.88%) |
| UA-2 | 41508000 (100.00%) | 1835 (0.00%) | 0 (0.00%) | 19060 (0.05%) | 2295545 (5.53%) | 31550 (0.08%) | 39160010 (94.34%) |
| UA-3 | 34452000 (100.00%) | 1439 (0.00%) | 0 (0.00%) | 13501 (0.04%) | 663517 (1.93%) | 9522 (0.03%) | 33764021 (98.00%) |
| UA-4 | 30737000 (100.00%) | 1282 (0.00%) | 0 (0.00%) | 11459 (0.04%) | 691781 (2.25%) | 14129 (0.05%) | 30018349 (97.66%) |
| UA-5 | 28222000 (100.00%) | 1238 (0.00%) | 0 (0.00%) | 130986 (0.46%) | 1145860 (4.06%) | 7359 (0.03%) | 26936557 (95.45%) |
| UA-6 | 37082000 (100.00%) | 1490 (0.00%) | 0 (0.00%) | 7668 (0.02%) | 751968 (2.03%) | 13195 (0.04%) | 36307679 (97.91%) |
| UA-7 | 28029000 (100.00%) | 1001 (0.00%) | 0 (0.00%) | 22326 (0.08%) | 1525781 (5.44%) | 23689 (0.08%) | 26456203 (94.39%) |
| UA-8 | 47179000 (100.00%) | 1811 (0.00%) | 0 (0.00%) | 8867 (0.02%) | 1076395 (2.28%) | 30322 (0.06%) | 46061605 (97.63%) |
| UA-9 | 29171000 (100.00%) | 543 (0.00%) | 0 (0.00%) | 16628 (0.06%) | 1145958 (3.93%) | 27708 (0.09%) | 27980163 (95.92%) |
| UA-10 | 28919000 (100.00%) | 695 (0.00%) | 0 (0.00%) | 9630 (0.03%) | 2557838 (8.84%) | 17813 (0.06%) | 26333024 (91.06%) |
| NSTEMI-1 | 49843000 (100.00%) | 826 (0.00%) | 0 (0.00%) | 6571 (0.01%) | 5212324 (10.46%) | 29755 (0.06%) | 44593524 (89.47%) |
| NSTEMI-2 | 41927000 (100.00%) | 1007 (0.00%) | 0 (0.00%) | 19076 (0.05%) | 5226096 (12.46%) | 22636 (0.05%) | 36658185 (87.43%) |
| NSTEMI-3 | 52786000 (100.00%) | 1715 (0.00%) | 0 (0.00%) | 17304 (0.03%) | 5255241 (9.96%) | 14383 (0.03%) | 47497357 (89.98%) |
| NSTEMI-4 | 48452000 (100.00%) | 917 (0.00%) | 0 (0.00%) | 8135 (0.02%) | 2761244 (5.70%) | 27311 (0.06%) | 45654393 (94.23%) |
| NSTEMI-5 | 41817000 (100.00%) | 772 (0.00%) | 0 (0.00%) | 22428 (0.05%) | 1167128 (2.79%) | 19275 (0.05%) | 40607397 (97.11%) |
| NSTEMI-6 | 43168000 (100.00%) | 462 (0.00%) | 0 (0.00%) | 23499 (0.05%) | 4647010 (10.76%) | 26940 (0.06%) | 38470089 (89.12%) |
| NSTEMI-7 | 49243000 (100.00%) | 331 (0.00%) | 0 (0.00%) | 70722 (0.14%) | 7564302 (15.36%) | 33785 (0.07%) | 41573860 (84.43%) |
| NSTEMI-8 | 43171723 (100.00%) | 823 (0.00%) | 0 (0.00%) | 27224 (0.06%) | 7706373 (17.85%) | 16541 (0.04%) | 35420762 (82.05%) |
| NSTEMI-9 | 51332000 (100.00%) | 1013 (0.00%) | 0 (0.00%) | 20211 (0.04%) | 1928446 (3.76%) | 24634 (0.05%) | 49357696 (96.15%) |
| NSTEMI-10 | 40134973 (100.00%) | 980 (0.00%) | 0 (0.00%) | 8428 (0.02%) | 1506541 (3.75%) | 16967 (0.04%) | 38602057 (96.18%) |
| STEMI-1 | 37884000 (100.00%) | 599 (0.00%) | 0 (0.00%) | 10970 (0.03%) | 1656254 (4.37%) | 15938 (0.04%) | 36200239 (95.56%) |
| STEMI-2 | 32041078 (100.00%) | 575 (0.00%) | 0 (0.00%) | 33709 (0.11%) | 2052781 (6.41%) | 10362 (0.03%) | 29943651 (93.45%) |
| STEMI-3 | 45684819 (100.00%) | 962 (0.00%) | 0 (0.00%) | 4532 (0.01%) | 1710441 (3.74%) | 13712 (0.03%) | 43955172 (96.21%) |
| STEMI-4 | 32825000 (100.00%) | 560 (0.00%) | 0 (0.00%) | 23814 (0.07%) | 6529313 (19.89%) | 10743 (0.03%) | 26260570 (80.00%) |
| STEMI-5 | 42398842 (100.00%) | 910 (0.00%) | 0 (0.00%) | 5051 (0.01%) | 1169192 (2.76%) | 10645 (0.03%) | 41213044 (97.20%) |
| STEMI-6 | 44943000 (100.00%) | 432 (0.00%) | 0 (0.00%) | 53764 (0.12%) | 3418062 (7.61%) | 23560 (0.05%) | 41447182 (92.22%) |
| STEMI-7 | 29796000 (100.00%) | 515 (0.00%) | 0 (0.00%) | 11838 (0.04%) | 3383036 (11.35%) | 11885 (0.04%) | 26388726 (88.56%) |
| STEMI-8 | 28193000 (100.00%) | 567 (0.00%) | 0 (0.00%) | 5129 (0.02%) | 860107 (3.05%) | 10326 (0.04%) | 27316871 (96.89%) |
| STEMI-9 | 28395000 (100.00%) | 261 (0.00%) | 0 (0.00%) | 19857 (0.07%) | 6780460 (23.88%) | 31917 (0.11%) | 21562505 (75.94%) |
| STEMI-10 | 34198000 (100.00%) | 503 (0.00%) | 0 (0.00%) | 8902 (0.03%) | 1228601 (3.59%) | 19826 (0.06%) | 32940168 (96.32%) |
