# Supplementary material for: Circulating microRNA expression profiling and bioinformatics analysis of patients with coronary artery disease by RNA sequencing
Source: J Clin Lab Anal. 2019 Sep 5;34(1):e23020. doi: 10.1002/jcla.23020 (PMC6977390; doi:10.1002/jcla.23020)
Supplement: Supplementary file 3 [file JCLA-34-e23020-s003.docx]

**TableS3 Gene oncology (GO) analysis of predicted target genes of differentially expressed microRNAs**

| GO ID (Lev2) | GO Term (Lev2) | GO Term (Lev1) | Gene Number | Total annotated genes |
| --- | --- | --- | --- | --- |
| GO:0005198 | structural molecule activity | Molecular Function | 510 | 19243 |
| GO:0004872 | receptor activity | Molecular Function | 1336 |  |
| GO:0003824 | catalytic activity | Molecular Function | 5948 |  |
| GO:0016530 | metallochaperone activity | Molecular Function | 6 |  |
| GO:0030545 | receptor regulator activity | Molecular Function | 63 |  |
| GO:0060089 | molecular transducer activity | Molecular Function | 1533 |  |
| GO:0030234 | enzyme regulator activity | Molecular Function | 1283 |  |
| GO:0016209 | antioxidant activity | Molecular Function | 63 |  |
| GO:0005488 | binding | Molecular Function | 14598 |  |
| GO:0000988 | protein binding transcription factor activity | Molecular Function | 623 |  |
| GO:0016247 | channel regulator activity | Molecular Function | 120 |  |
| GO:0005215 | transporter activity | Molecular Function | 1275 |  |
| GO:0001071 | nucleic acid binding transcription factor activity | Molecular Function | 1269 |  |
| GO:0045182 | translation regulator activity | Molecular Function | 36 |  |
| GO:0032991 | macromolecular complex | Cellular Component | 5444 |  |
| GO:0031012 | extracellular matrix | Cellular Component | 427 |  |
| GO:0045202 | synapse | Cellular Component | 585 |  |
| GO:0044464 | cell part | Cellular Component | 16571 |  |
| GO:0005576 | extracellular region | Cellular Component | 3883 |  |
| GO:0031974 | membrane-enclosed lumen | Cellular Component | 5173 |  |
| GO:0005623 | cell | Cellular Component | 16571 |  |
| GO:0044456 | synapse part | Cellular Component | 547 |  |
| GO:0016020 | membrane | Cellular Component | 8962 |  |
| GO:0043226 | organelle | Cellular Component | 13471 |  |
| GO:0044421 | extracellular region part | Cellular Component | 3883 |  |
| GO:0044420 | extracellular matrix part | Cellular Component | 197 |  |
| GO:0030054 | cell junction | Cellular Component | 803 |  |
| GO:0009295 | nucleoid | Cellular Component | 42 |  |
| GO:0044423 | virion part | Cellular Component | 29 |  |
| GO:0019012 | virion | Cellular Component | 29 |  |
| GO:0044425 | membrane part | Cellular Component | 7239 |  |
| GO:0044422 | organelle part | Cellular Component | 9331 |  |
| GO:0000003 | reproduction | Biological Process | 1303 |  |
| GO:0008152 | metabolic process | Biological Process | 11837 |  |
| GO:0048519 | negative regulation of biological process | Biological Process | 4558 |  |
| GO:0032501 | multicellular organismal process | Biological Process | 7275 |  |
| GO:0022610 | biological adhesion | Biological Process | 1172 |  |
| GO:0040007 | growth | Biological Process | 915 |  |
| GO:0002376 | immune system process | Biological Process | 3104 |  |
| GO:0051704 | multi-organism process | Biological Process | 1757 |  |
| GO:0001906 | cell killing | Biological Process | 171 |  |
| GO:0044699 | single-organism process | Biological Process | 13424 |  |
| GO:0032502 | developmental process | Biological Process | 6032 |  |
| GO:0051179 | localization | Biological Process | 6200 |  |
| GO:0048511 | rhythmic process | Biological Process | 258 |  |
| GO:0051234 | establishment of localization | Biological Process | 5119 |  |
| GO:0023052 | signaling | Biological Process | 6373 |  |
| GO:0050896 | response to stimulus | Biological Process | 8666 |  |
| GO:0048518 | positive regulation of biological process | Biological Process | 5316 |  |
| GO:0009987 | cellular process | Biological Process | 15928 |  |
| GO:0071840 | cellular component organization or biogenesis | Biological Process | 6217 |  |
| GO:0050789 | regulation of biological process | Biological Process | 11165 |  |
| GO:0022414 | reproductive process | Biological Process | 1168 |  |
| GO:0040011 | locomotion | Biological Process | 1699 |  |
| GO:0065007 | biological regulation | Biological Process | 11681 |  |
