# Supplementary material for: Circulating microRNA expression profiling and bioinformatics analysis of patients with coronary artery disease by RNA sequencing
Source: J Clin Lab Anal. 2019 Sep 5;34(1):e23020. doi: 10.1002/jcla.23020 (PMC6977390; doi:10.1002/jcla.23020)
Supplement: Supplementary file 4 [file JCLA-34-e23020-s004.docx]

**TableS4 The top 20 KEGG pathways**

| Pathway term | Rich factor | P Value | Gene number |
| --- | --- | --- | --- |
| Endocytosis | 0.9962 | 0.2807 | 259 |
| Pathways in cancer | 0.9849 | 0.2826 | 391 |
| Focal adhesion | 1.0000 | 0.2942 | 203 |
| Axon guidance | 1.0000 | 0.3097 | 176 |
| Rap1 signaling pathway | 0.9905 | 0.3248 | 209 |
| Proteoglycans in cancer | 0.9902 | 0.3287 | 203 |
| AMPK signaling pathway | 1.0000 | 0.3446 | 125 |
| Hippo signaling pathway | 0.9935 | 0.3449 | 153 |
| Lysosome | 1.0000 | 0.3462 | 123 |
| Hepatitis B | 0.9932 | 0.3511 | 145 |
| PI3K-Akt signaling pathway | 0.9737 | 0.3533 | 333 |
| Signaling pathways regulating pluripotency of stem cells | 0.9930 | 0.3543 | 141 |
| Insulin signaling pathway | 0.9928 | 0.3567 | 138 |
| Osteoclast differentiation | 0.9924 | 0.3626 | 131 |
| Ras signaling pathway | 0.9781 | 0.3671 | 223 |
| Inflammatory mediator regulation of TRP channels | 1.0000 | 0.3675 | 98 |
| Endocrine resistance | 1.0000 | 0.3684 | 97 |
| Glycerophospholipid metabolism | 1.0000 | 0.3703 | 95 |
| Sphingolipid signaling pathway | 0.9917 | 0.3723 | 120 |
| Leukocyte transendothelial migration | 0.9915 | 0.3751 | 117 |
